# Distinct Exocrine Glands Contribute to the Chemical Polymorphism Across Developmental Stages and Sexes in *Riptortus pedestris*

**DOI:** 10.3390/insects17060568

**Published:** 2026-05-29

**Authors:** Sylvestre T. O. Kelehoun, Lian-Ying Peng, Shao-Hang Yang, Lai Wei, Ya-Nan Zhang, Ming-Sheng Yang, Kai Li, Hao Xu

**Affiliations:** 1 State Key Laboratory of Agricultural and Forestry Biosecurity, College of Plant Protection, Nanjing Agricultural University, Nanjing 210095, China; 2023102292@stu.njau.edu.cn (S.T.O.K.); 2023102096@stu.njau.edu.cn (L.-Y.P.); 2021102095@stu.njau.edu.cn (S.-H.Y.); weilai@stu.njau.edu.cn (L.W.); 2 Key Laboratory of Soybean Disease and Pest Control, Ministry of Agriculture and Rural Affairs, Nanjing Agricultural University, Nanjing 210095, China; 3 Anhui Engineering Research Center for Green Production Technology of Drought Grain Crops, College of Life Sciences, Huaibei Normal University, Huaibei 235000, China; ynzhang_insect@163.com; 4 College of Life Science and Agronomy, Zhoukou Normal University, Zhoukou 466001, China; yms-888@163.com; 5 Soybean Research Institute, Nanjing Agricultural University, Nanjing 210095, China

**Keywords:** agrochemicals, chemical ecology, odor gland, pest management, soybean, sternal gland

## Abstract

The bean bug *Riptortus pedestris* is one of the principal soybean pests throughout East Asia. Males release an aggregation pheromone consisting of three compounds—(*E*)-2-hexenyl (*Z*)-3-hexenoate (E2HZ3H), (*E*)-2-hexenyl (*E*)-2-hexenoate (E2HE2H) and myristyl isobutyrate (MI)—in a ratio of 1:5:1. Although pheromone-based baits have been extensively deployed to control the pest for the past three decades, the glands responsible for producing these volatiles have remained unidentified. Our investigations revealed that the metathoracic glands (MTGs) of males synthesized the two pheromonal esters, E2HZ3H and E2HE2H, along with a suite of auxiliary compounds: (*E*)-2-hexenal, hexanoic acid, (*E*)-3-hexenoic acid, (*E*)-2-hexenoic acid, (*E*)-2-octenal, and (*E*)-2-hexenyl hexanoate (E2HH). Female MTGs were morphologically similar to those of males and generated the same auxiliary compounds, but lacked the capacity to produce the pheromonal esters E2HZ3H and E2HE2H. MI was released exclusively from the male abdominal sternites. Its quantity was nearly uniform across all sternal segments, implying secretion from an epidermal glandular patch (GP) that was evenly distributed over the sternites. In nymphs, dorsal abdominal glands (DAGs) emitted several volatiles—including (*E*)-2-hexenal, 4-oxo-(*E*)-2-hexenal, (*E*)-2-octenal, and (*E*)-2-octenoic acid—some of which are characteristic defensive compounds of heteropterans. Thus, our study clarifies how sex- and stage-specific glands contribute to the chemical polymorphism observed in *R. pedestris*.

## Simple Summary

As a notorious stink bug pest, the bean bug *Riptortus pedestris* (Hemiptera: Alydidae) produces a variety of semiochemicals across its life stages. In this study, we integrated morphological examinations with chemical analyses to elucidate how males, females, and nymphs utilize specific odor glands to produce both pheromonal and auxiliary volatiles. Our findings resolve a long-standing gap in the chemical ecology of this economically important pest, presenting a novel model of sexually dimorphic and ontogenetically regulated glandular architecture in heteropteran species.

## 1. Introduction

Heteropterans are renowned for releasing complex, pungent chemicals that fulfill a variety of ecological roles [1,2]. Both immature and adult stages possess well-developed exocrine glands that synthesize and emit these compounds. The main odor-producing structures include the dorsal abdominal glands (DAGs), metathoracic glands (MTGs), and glandular patches (GPs) located on the abdominal sternites [1,2]. Identifying the precise sources of pheromone emissions is essential for understanding where chemical signals are produced, stored, and released, thereby shedding light on the functional significance and evolutionary dynamics of heteropteran odor glands [1].

Heteropteran nymphs primarily employ DAGs to synthesize and release defensive compounds such as (*E*)-2-hexenal, (*E*)-2-octenal, 4-oxo-(*E*)-2-hexenal etc., many of which contain an *α*,*β*-unsaturated bioactive functional group [3,4,5]. In contrast, adult heteropterans possess a much more diverse repertoire of odor glands—including DAGs, MTGs and GPs—that vary widely among taxa and even between sexes [1,2,6,7]. Female semiochemicals that are often released by MTGs are believed to defend against natural enemies, and those compounds comprise alkanes, *α*,*β*-unsaturated compounds, esters and terpenoids, etc. [2,8,9]. Males may emit specific semiochemicals from the same gland (e.g., MTGs) or from additional glands such as DAGs or GPs [1,2,8]. In many species, these male-specific volatiles have been demonstrated to function as aggregation or sex pheromones [4,9,10,11]. The chemical classes of male-specific compounds are especially diverse, encompassing terpenoids, branched fatty acid-derived molecules, compounds with benzene rings, etc. [1,11,12].

The bean bug, *Riptortus pedestris* (Hemiptera: Alydidae), is a major agricultural pest that is widely distributed throughout East Asia. It causes severe damage to numerous legume crops, including soybean (*Glycine max*), faba bean (*Vicia faba*), pea (*Pisum sativum*), and mung bean (*Vigna radiata*) [12,13,14]. In soybean, *R. pedestris* is especially notorious for inducing “stay-green syndrome” a disorder characterized by delayed leaf and stem senescence, irregular pod development, and high rates of seed abortion [15,16,17].

In *R. pedestris*, the composition of volatile compounds changes markedly across developmental stages and between sexes. As in many other heteropterans, nymphs emit large quantities of defensive (*E*)-2-alkenals, notably (*E*)-2-hexenal, 4-oxo-(*E*)-2-hexenal, and (*E*)-2-octenal [5,18]. Adult bugs, in contrast, produce a suite of adult-specific carboxylic acids—hexanoic acid, (*E*)-3-hexenoic acid, and (*E*)-2-hexenoic acid—together with their possible corresponding esters, such as (*E*)-2-hexenyl hexanoate (E2HH), (*E*)-2-hexenyl (*Z*)-3-hexenoate (E2HZ3H), and (*E*)-2-hexenyl (*E*)-2-hexenoate (E2HE2H) [18]. Male adults release three compounds that are absent in females: E2HZ3H, E2HE2H, and myristyl isobutyrate (MI) [9,18]. These three volatiles function as the species’ aggregation pheromone in a characteristic 1:5:1 ratio [9], and have been widely employed in field traps to capture both adults and nymphs throughout East Asia [19,20,21,22].

Despite the practical importance, the biosynthetic pathways and glandular sources of these semiochemicals in *R. pedestris* remain poorly understood. In the present study, we integrated morphological examinations with chemical analyses to elucidate how different glandular systems contribute to the observed chemical polymorphism across developmental stages and between sexes in *R. pedestris*.

## 2. Methodology

### 2.1. Insects

Adult *R. pedestris* were captured from wild populations using pheromone traps (Pherobio, Beijing, China) in Nanjing, Jiangsu Province, East China, during the summer of 2025. The collected bugs were then maintained in an incubator set to 25 °C, a 16 h:8 h light–dark photoperiod, and 70% relative humidity. Individuals were housed in 30 × 30 × 30 cm mesh cages and provided with soybean seeds and 3-week-old soybean seedlings (cultivar Zhonghuang 13) as food and water sources, respectively. For the experiments, fourth-instar nymphs and sexually mature adults (10–15 days old) were used as described below.

### 2.2. Insect Dissection, Organ Collection and Solvent Extraction

Ten fourth-instar nymphs, as well as ten male and ten female adults of *R. pedestris*, were killed by freezing at −20 °C for about 10 min. Each specimen was then dissected under a stereomicroscope into three main parts: head, thorax (including all legs and wings whenever possible), and abdomen. DAGs of nymphs and MTGs of adults were then collected. Individual tissues or isolated glands from nymphs, male or female adults (10 individuals each) were placed in 1.5 mL glass vials (Agilent Technologies), and 100–200 µL of analytical-grade dichloromethane (Macklin, Shanghai, China, 99%) was added. Vials were vortexed for about 30 s using a Vortex-Genie 2 mixer (Scientific Industries, Bohemia, NY, USA). Extraction proceeded for 10 min at ambient temperature. The liquid supernatant was drawn off with a glass syringe and stored at −20 °C until chemical analysis. The experiment consisted of three replicates.

During preliminary comparisons of volatile profiles from different male body sections, MI was exclusively detected in extracts from the abdomens. To pinpoint its precise source, the male abdomens (from 10 individuals) were further dissected into the following organs: midgut (including gastric caeca and Malpighian tubules), hindgut, reproductive organs, fat bodies and integument (epidermis and cuticle). Each organ was extracted and stored using the same protocol described above. For finer spatial resolution, the abdominal integument was divided into individual dorsal (tergites) and ventral (sternites) sclerites—five tergites and five sternites—and the chemicals of each segment were extracted separately. The experiment consisted of three replicates.

### 2.3. Light Microscopy

*R. pedestris* were killed by freezing at −20 °C for approximately 10 min. Fifth-instar nymphs and adults of both sexes (about ten individuals per group) were used for morphological light microscopy. Nymphal DAGs were isolated under a stereomicroscope, whereas the ventral thoracic sternites of adults were removed to expose the MTG complex. All dissected samples were immediately imaged using a Nikon SMZ25 stereomicroscope (Nikon Corporation, Tokyo, Japan).

### 2.4. Scanning Electron Microscopy (SEM) Sample Preparation

*R. pedestris* gland specimens (about five individuals per group) were fixed in 0.1 M phosphate-buffered saline (PBS, pH 7.0) containing 2.5% glutaraldehyde. After vacuum infiltration until the samples sank, they were stored at 4 °C overnight. The specimens were then washed three times with 0.1 M PBS (pH 7.0) for 15 min each to remove residual fixative. Subsequently, the specimens were dehydrated by immersing them in a graded ethanol series (50%, 70%, 80% and 90% *v*/*v*) for 15 min per step, followed by five successive washes in 100% ethanol (30 min each). The dehydrated tissues were subsequently infiltrated with tert-butanol (five exchanges, 30 min each). After infiltration, the samples were freeze-dried, mounted on aluminum stubs with an adhesive carbon strip, and sputter-coated with a thin layer of gold. Imaging was performed on a Hitachi Regulus 8100 scanning electron microscope (Hitachi High-Tech Corporation, Hitachinaka, Japan).

### 2.5. Gas Chromatography-Mass Spectrometry (GC-MS) Analyses

Samples were analyzed using an Agilent 8890 gas chromatograph (GC) coupled with an Agilent 5977B mass spectrometer (MS) detector (Agilent Technologies Inc., Palo Alto, CA, USA). For each sample, 2 µL of extract was injected into the GC column using a 10% split mode. The injector temperature was set at 250 °C. Helium was used as the carrier gas at a constant flow rate of 1 mL/min through a non-polar HP-5ms column (30 m × 0.25 mm ID, 0.25 µm film thickness; Agilent Technologies). The oven temperature was initially set at 40 °C and held for 7 min, then increased at a rate of 5 °C/min to 55 °C, followed by a ramp of 15 °C/min to 250 °C, where it was held for 2 min. Compounds were identified based on their mass spectra by comparison with the NIST Mass Spectral Library database (Version 2.4). Their identities had been identified previously [18], and here were confirmed by comparing the GC retention times and mass spectra with commercial synthetic standards. Two internal standards (n-octane and nonyl acetate; 200 ng each in 10 µL of dichloromethane) were added to each sample as references for compound quantification.

### 2.6. Statistical Analysis

The amounts of compounds (ng) across treatments (*n* = 3), including body sections (heads, thoraxes, abdomens), segments of abdominal sternites (or tergites), and the glandular tissues (*n* = 3), were first compared using the generalized linear model (GLM) with a Gaussian error distribution. When the model indicated significant differences appeared, the estimated marginal means were calculated and pairwise comparisons were performed using the *emmeans* package [23] in R 4.5.2.

## 3. Results

**Figure 1:**
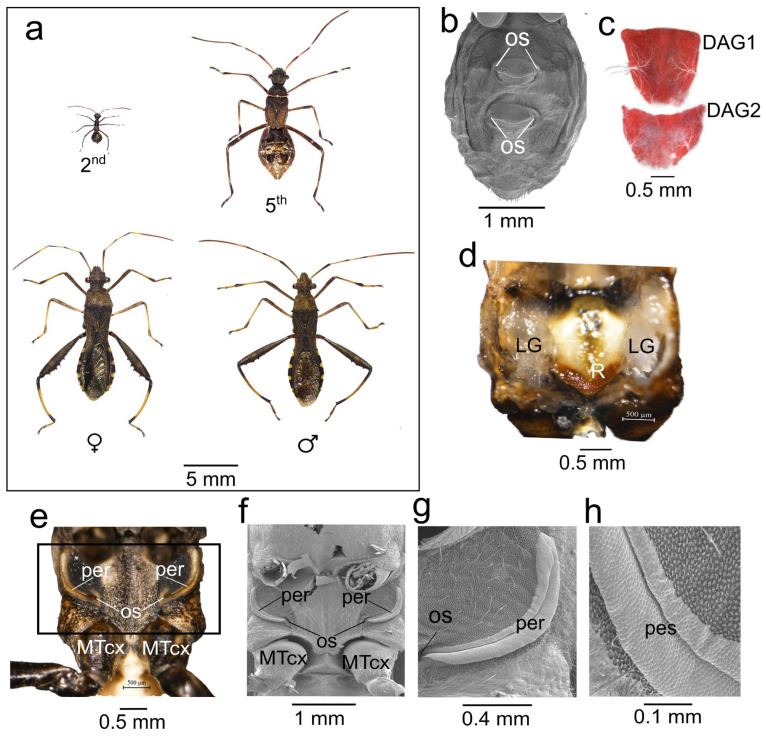
Morphology of *Riptortus pedestris* and the odor glands. (**a**) The dorsal view of the second-instar nymph, the fifth-instar nymph, the female and the male. (**b**) Scanning electron micrograph (SEM) of the dorsal abdomen of an immature showing ostioles (os). (**c**) Dorsal abdominal glands 1 and 2 (DAG 1 & 2) from a fifth-instar nymph. (**d**) Internal view of the metathoracic glandular system, showing the lateral glands (LG) and the reservoir (R). (**e**) External view of the metathoracic region (outlined by a rectangle), showing the ostioles (os) for scent release and the peritremes (per) for secretion dispersion. (**f**) SEM of the external metathoracic region (outlined by a rectangle), detailing the ostioles (os) and peritremes (per). (**g**) SEM close-up of an ostiole (os) and a peritreme (per). (**h**) SEM detailing the surface structure of a peritreme surface (pes). MTcx: Metathoracic coxa.

Figure 1 illustrates the morphological differences between adults and nymphs of *R. pedestris*. The second-instar nymphs display ant mimicry (Figure 1a). In immature stages, the odor-producing structures are DAGs (Figure 1b,c), whereas adults possess MTGs (Figure 1d–h). Two DAGs are arranged sequentially along the abdominal tergites of nymphs: DAG 1 resides between tergites 4 and 5, and DAG 2 lies between tergites 5 and 6 (Figure 1b,c). Each DAG terminates in a pair of ostioles situated at the anterior margin of its respective inter-tergal region (Figure 1b).

An MTG is situated ventrally on the metathorax of both male and female adults (Figure 1e). Each MTG consists of a pair of lateral glands (LGs) that appear to be linked to a central orange-colored reservoir (Figure 1d). The glandular complex opens via a pair of ostioles located adjacent to the metathoracic coxae (Figure 1e–g). From each ostiole, a peritreme extends outward as a curved, comma-shaped structure, presumably facilitating the diffusion of the gland secretion (Figure 1e–h). No notable morphological differences were observed between the MTGs of males and females.

**Figure 2:**
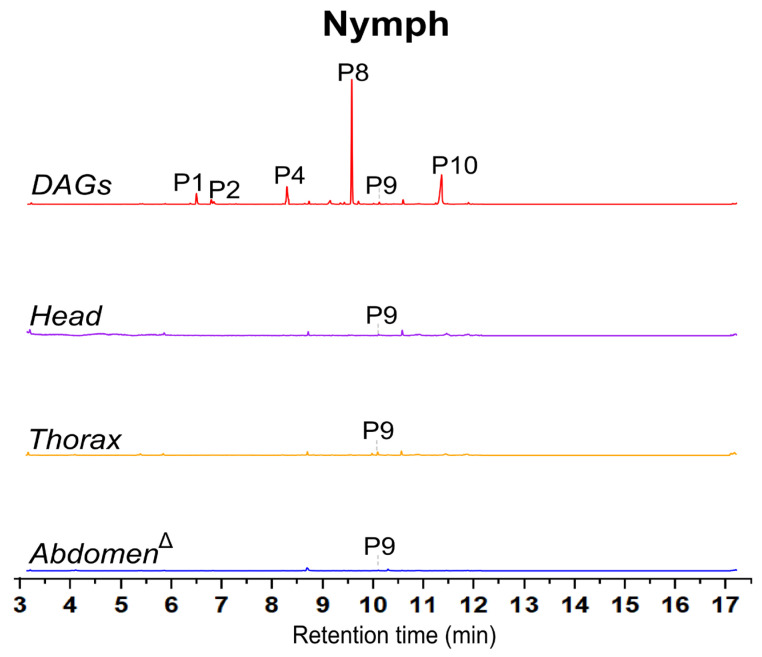
Typical chromatographs of the solvent extracts of dorsal abdominal glands (DAGs), heads, thoraxes and abdomens of fourth-instar nymphs of *Riptortus pedestris*. Identified compounds: P1, (*E*)-2-hexenal; P2, (*E*)-2-hexenol; P4, 4-oxo-(*E*)-2-hexenal; P8, (*E*)-2-octenal; P9, nonanal; P10, (*E*)-2-octenoic acid. (Δ): The abdomens were free of DAGs.

Nymphal semiochemicals were predominantly produced by the DAGs (Figure 2; Table 1, *n* = 3), comprising the defensive *α*,*β*-unsaturated aldehydes (*E*)-2-hexenal, 4-oxo-(*E*)-2-hexenal, and (*E*)-2-octenal, together with their putative derivatives such as (*E*)-2-hexenol and (*E*)-2-octenoic acid (Figure 2; Table 1). In contrast, nonanal was distributed uniformly across all body sections (heads, thoraxes, and abdomens) and was not confined to the DAGs (Figure 2; Table 1).

**Figure 3:**
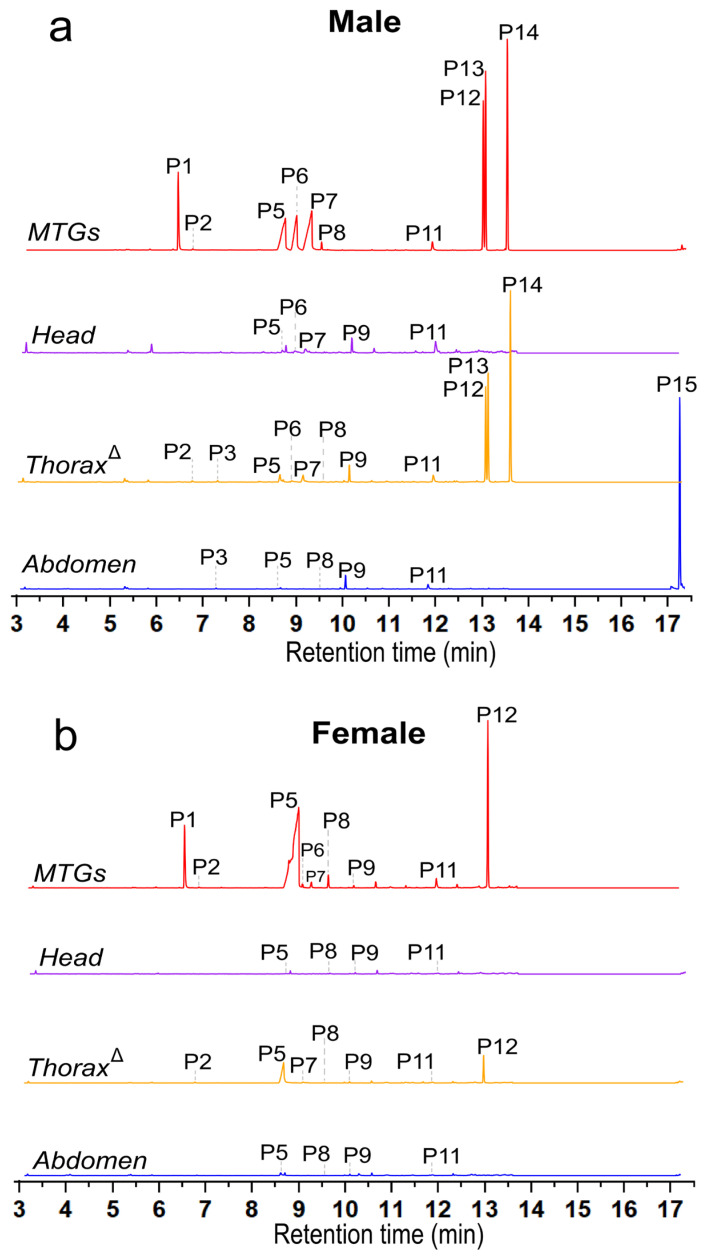
Typical chromatographs of the solvent extracts of metathoracic glands (MTGs), heads, thoraxes and abdomens of *Riptortus pedestris* males (**a**) and females (**b**). Identified compounds: P1, (*E*)-2-hexenal; P2, (*E*)-2-hexenol; P3, heptanal; P5, hexanoic acid; P6, (*E*)-3-hexenoic acid; P7, (*E*)-2-hexenoic acid; P8, (*E*)-2-octenal; P9, nonanal; P11, nonanoic acid; P12, (*E*)-2-hexenyl hexanoate (E2HH); P13, (*E*)-2-hexenyl (*Z*)-3-hexenoate (E2HZ3H); P14, (*E*)-2-hexenyl (*E*)-2-hexenoate (E2HE2H); and P15, myristyl isobutyrate (MI). (Δ): The thoraxes were free of MTGs.

In adults, MTGs were the principal source of semiochemicals, including (*E*)-2-hexenal, (*E*)-2-hexenol, hexanoic acid, (*E*)-3-hexenoic acid, (*E*)-2-hexenoic acid, (*E*)-2-octenal, and the ester compounds E2HH, E2HZ3H, and E2HE2H (Figure 3; Table 2, *n* = 3). Among these, E2HZ3H and E2HE2H were male-specific (Figure 3a). Substantial amounts of MTG-derived compounds—particularly hexanoic acid, E2HZ3H, E2HE2H, and E2HH—were also detected in extracts of thoracic tissue that had been dissected free of MTGs (Figure 3). This suggests that these volatiles had diffused from the MTGs into the comma-shaped peritremes before gland removal (Figure 1e–h). The principal pheromonal component MI, which is known to be attractive to both adults and nymphs in field assays [24,25], was detected exclusively in extracts of male abdomens (Figure 3a; Table 2). In addition to qualitative differences, quantitative variations between the sexes were evident (Table 2, *n* = 3). For instance, (*E*)-3-hexenoic acid and (*E*)-2-hexenoic acid were present in significantly higher amounts in male MTGs than in female MTGs. As observed in nymphs, several adult compounds were not confined to a particular body region or gland. For example, nonanoic acid and nonanal were distributed uniformly across heads, thoraxes, abdomens and MTGs (Figure 3; Table 2, *n* = 3).

**Figure 4:**
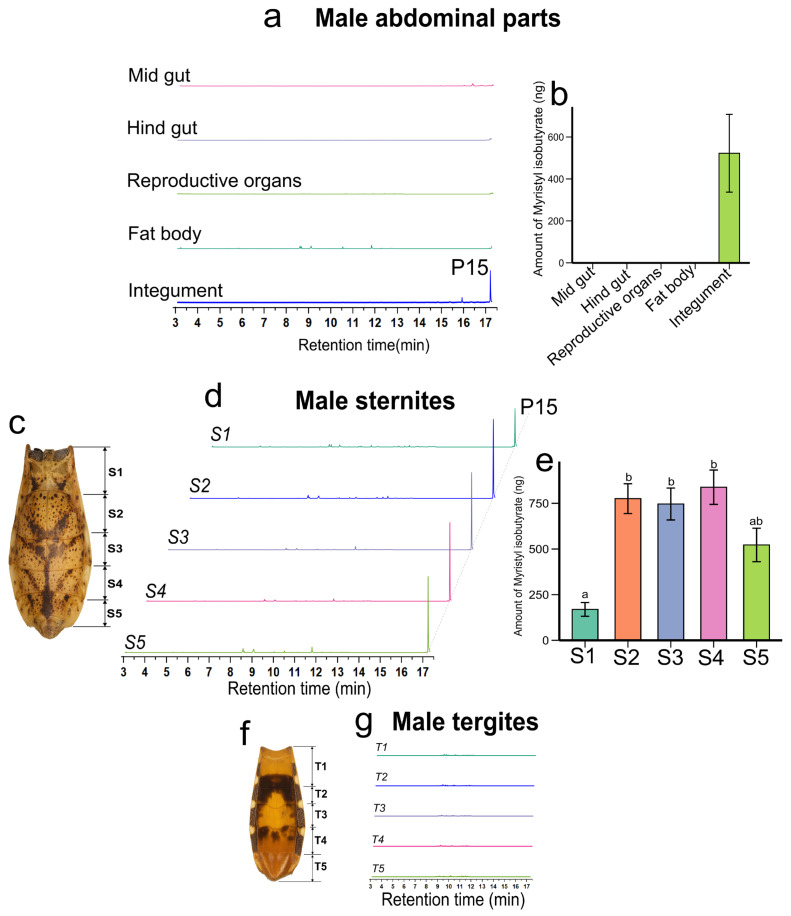
Chemical profiles (**a**) and corresponding amounts (**b**) of myristyl isobutyrate (MI, P15) in different abdominal tissues of *Riptortus pedestris* males, including the midgut with gastric caeca and Malpighian tubules, hindgut, reproductive organs, fat body and integument. Ventral view of the abdomen showing the five abdominal sternites (S1–S5) (**c**). Chemical profiles (**d**) and corresponding amounts (**e**) of MI across the five abdominal sternites. Dorsal view of the abdomen showing the five abdominal tergites (T1–T5) (**f**). Chemical profiles of the five abdominal tergites (**g**). Pairwise comparisons were performed on the amounts of MI (mean ± SEM, ng; *n* = 3) among the different sternites. Different lowercase letters indicate significant differences (*p* < 0.05).

Additional analyses revealed that MI was detected exclusively in extracts of the abdominal integument and was absent from internal tissues such as the midgut (including gastric caeca and Malpighian tubules), hindgut, reproductive organs, and fat bodies (Figure 4a,b). When the male abdomen was dissected into its individual sternal and tergal segments, MI was recovered only from the sternites; none of the tergites yielded detectable MI (Figure 4c–g). Moreover, the amount of MI was roughly comparable across sternite segments 2–5, whereas the first sternite also contained MI but at a somewhat lower concentration (Figure 4c–e, *n* = 3). These findings suggest that MI is secreted by an epidermal glandular patch (GP) that is broadly, though not uniformly, distributed across the male sternites.

## 4. Discussion

*R. pedestris* has garnered significant attention from entomologists in East Asia for several decades [17,20,21,22,26]. For example, Leal et al. (1995) first identified an aggregation pheromone released by males, consisting of E2HZ3H, E2HE2H and MI in a 1:5:1 ratio; this blend attracts both sexes and nymphs [9]. Since then, the pheromone mixture has been widely employed for population monitoring and mass-trapping of the pest in Japan, Korea, and China [19,20,21]. Our previous work demonstrated that volatile profiles differ according to sex and developmental stage [18]. However, the specific glandular organs responsible for the biosynthesis and release of these pheromonal compounds and other semiochemicals have yet to be identified. By integrating chemical and morphological analyses of a range of body tissues, we have now shown that three distinct odor glands—DAGs, MTGs and ventral abdominal GPs—are responsible for the chemical polymorphism observed in *R. pedestris*.

***DAGs and MTGs underlie the scent differences between nymphs and adults***—We found that DAGs are the sole scent glands present during the nymphal stages of *R. pedestris*. DAGs are common in stink bug nymphs and are retained in adults of many species [27,28,29]. In nymphs, DAG exudates are typically dominated by defensive C_6_ and C_8_ *α*,*β*-unsaturated aldehydes [2,5,30], such as the volatiles detected in *R. pedestris* nymphs: (*E*)-2-hexenal, (*E*)-2-octenal, and 4-oxo-(*E*)-2-hexenal (Figure 2; Table 1) [5]. These compounds likely function as anti-predator chemicals against arthropod enemies (e.g., spiders and mantids) [2,5,31]. In addition to the aldehydes, the *α*,*β*-unsaturated carboxylic acid (*E*)-2-octenoic acid was also identified. This molecule has been reported from males of the congener *R. serripes* and from nymphs of the lygaeid bug *Geocoris punctipes* [32,33]. Its *α*,*β*-unsaturated functional group suggests a defensive role analogous to that of the aldehydes. However, DAGs’ secretions could sometimes promote the aggregation of newly hatched nymphs, as reported previously [29,34].

Adult heteropterans possess several odor-gland types, notably MTGs, DAGs and GPs [2]. Only the MTGs are active in male and female *R. pedestris*, while DAGs are absent in the adult stage. The MTGs primarily secrete short-chain carboxylic acids: hexanoic acid, (*E*)-3-hexenoic acid, (*E*)-2-hexenoic acid, and three ester derivatives: E2HH, E2HZ3H and E2HE2H. Ester formation likely depends on the availability of C_6_ carboxylic acids and C_6_ alcohols. For example, E2HE2H may arise from the enzymatic condensation of (*E*)-2-hexenoic acid with (*E*)-2-hexenol, whereas E2HH could be generated from hexanoic acid combined with (*E*)-2-hexenol.

In adults, the scent repertoire serves both defensive and aggregative (or sexual) functions. For instance, the non-sex-specific ester E2HH, emitted from the MTGs of *R. pedestris*, attracts conspecific nymphs and may guide them to food resources where adults are feeding [18]. Moreover, E2HH enhances the attractiveness of the male aggregation pheromone to conspecific adults and has even captured males when deployed alone in field traps [35]. Simultaneously, MTG secretions of heteropteran females are also interpreted as defensive compounds against predators [29,36,37]. By contrast, males supplement the basic defensive blend with additional volatiles that fulfill sexual or aggregation roles, either via their MTGs or through auxiliary glandular structures (discussed below).

***Adult odor glands and their roles in sexual dimorphism***—Although the external morphology of the MTGs is indistinguishable between male and female *R. pedestris*, their chemical outputs differ markedly. Male MTGs produce additional compounds such as E2HZ3H and E2HE2H, and they release greater quantities of putative precursors like (*E*)-3-hexenoic acid and (*E*)-2-hexenoic acid. This male-biased enrichment of volatile blends is a common pattern among stink bugs, where males emit aggregation or sex pheromones that attract conspecific adults and nymphs for purposes ranging from food sharing to mate location [1,38]. The MTG secretions of *R. pedestris* display a clear chemical sexual dimorphism, a pattern also documented in other heteropteran species [32,39]. 

Nonetheless, in many taxa, MTG chemistry is sexually monomorphic, and males rely on auxiliary glands to synthesize aggregation or sex pheromones. This includes predatory species such as *Arma custos* [29], *Podisus maculiventris* [11], and *Eocanthecona furcellata* [4], as well as some phytophagous species, such as *Amblypelta lutescens lutescens* [32] and *Dysdercus intermedius* [40]. In these examples, male-specific glands such as GPs and/or DAGs complement the MTGs, furnishing the additional volatiles required for sexual communication and aggregation [4,29,41].

In adult *R. pedestris*, we did not detect DAGs. Instead, the male-specific pheromonal component MI is released from GPs located on the abdominal sternites. Earlier work on stink bugs—predominantly from the family Pentatomidae—has also shown that GPs occur only on the ventral abdominal segments of males [1,4,41,42]. The volatiles emitted by GPs are primarily involved in food-location and mating stimulation [1,43], supporting the notion that male heteropterans usually bear the responsibility for producing aggregation and sex pheromones. Within the subfamily Asopinae, GPs are concentrated on abdominal sternal segments 5–7 and exhibit a characteristic morphology: a cluster of glandular pores surrounded by a group of setae that functions as a conduit for secretions to reach the exterior [41]. However, as an alydid, male *R. pedestris* releases MI from all abdominal sternal segments, and we did not observe these grouped setae. Although the secretion probably occurs on every segment, the quantity varies among them, likely because the cuticular surface area differs, with the first sternite being relatively smaller (see Figure 4c). This pattern suggests that the *R. pedestris* GPs are evenly distributed across the abdominal ventral cuticle.

## 5. Conclusions

By dissecting the odor-gland systems of nymphs, males and females of *R. pedestris*, we have clarified how each life stage and sex generates its characteristic volatile blend. These findings deepen our understanding of the diversity and evolutionary plasticity of glandular architectures in heteropterans, highlighting how they diverge across developmental stages and between the sexes.

## Figures and Tables

**Table 1:** The amounts of volatile compounds (mean ± SE, in ng) in nymphs.

Compounds	RT (min)	Nymph				Tissue Effect
		Head	Thorax	DAGs	Abdomen ^Δ^	
(*E*)-2-Hexenal	6.55	n.d.	n.d.	**19 ± 7**	n.d.	-
(*E*)-2-Hexenol	6.84	n.d.	n.d.	**7 ± 3**	n.d.	-
4-oxo-(*E*)-2-hexenal	8.29	n.d.	n.d.	**51 ± 6**	n.d.	-
(*E*)-2-Octenal	9.57	n.d.	n.d.	**647 ± 169**	n.d.	-
Nonanal	10.11	2 ± 0.11 a**#**	**7 ± 0.30 c**	3 ± 0.41 b	2 ± 0.20 a	F_3,8_ = 87.55; *p* < 0.001
(*E*)-2-Octenoic acid	11.30	n.d.	n.d.	**170 ± 20**	n.d.	-

RT: Retention time; DAGs: dorsal abdominal glands. Data were analyzed using generalized linear models (GLMs) with a Gaussian error distribution. For each compound, comparisons were performed among tissues (head, thorax, DAGs, and abdomen). Estimated marginal means were then calculated, and pairwise comparisons were conducted using the *emmeans* package in R. (#): Different lowercase letters indicate statistical differences among tissues for each compound (*p* < 0.05). Bold numbers indicate the highest amount of the compound in a specific tissue. n.d. indicates that the compound was not detected. (Δ): The DAGs had been removed for the abdomens.

**Table 2:** The amounts of volatile compounds (mean ± SE, in ng) in adult *Riptortus pedestris*.

		Male				Female				
Compounds	RT (min)	Head	Thorax ^Δ^	MTGs	Abdomen	Head	Thorax ^Δ^	MTGs	Abdomen	Tissue Effect
**(*E*)-2-hexenal**	6.55	n.d.	n.d.	**202 ± 78 a#**	n.d.	n.d.	n.d.	**146 ± 44 a**	n.d.	F_1,4_ = 0.09; *p* = 0.771
**(*E*)-2-hexenol**	6.84	n.d.	7 ± 0.5 a	10 ± 2 a	n.d.	n.d.	16.25 ± 10.11 a	3 ± 2 a	n.d.	F_3,8_ = 1.78; *p* = 0.228
**heptanal**	7.35	n.d.	4 ± 1 a	n.d.	4 ± 3 a	n.d.	n.d.	n.d.	n.d.	F_1,4_ = 0.34; *p* = 0.589
**hexanoic acid**	8.66	8 ± 3 a	34 ± 5 a	**1839 ± 727 b**	11 ± 4 a	9 ± 4 a	**349 ± 69 b**	**2046 ± 416 b**	11 ± 3 a	F_7,16_ = 37.58; *p* < 0.001
**(*E*)-3-hexenoic acid**	8.9	3 ± 1 a	4 ± 2 a	**1111 ± 363 b**	n.d.	n.d.	1 ± 0.23 a	trace	n.d.	F_4,10_ = 62.59; *p* < 0.001
**(*E*)-2-hexenoic acid**	9.14	13 ± 3 a	28 ± 12 a	**2108 ± 722 b**	n.d.	n.d.	17 ± 7 a	68 ± 39 a	n.d.	F_4,10_ = 17.78; *p* < 0.001
**(*E*)-2-octenal**	9.57	n.d.	2 ± 1 a	**24 ± 1 b**	trace	trace	1.39 ± 0.38 a	**23 ± 5 b**	trace	F_6,14_ = 33.71; *p* < 0.001
**nonanal**	10.11	5 ± 2 ab	**35 ± 11 b**	2 ± 1 a	40 ± 33 ab	2 ± 0.27 a	5 ± 0.89 ab	4 ± 1 ab	5 ± 1 ab	F_7,16_ = 4.17; *p* = 0.009
**nonanoic acid**	11.85	12 ± 2 ab	30 ± 7 abc	**101 ± 44 c**	29 ± 15 abc	6 ± 2 a	17 ± 7 ab	34 ± 4 bc	11 ± 1 ab	F_7,16_ = 5.30; *p* < 0.001
**E2HH**	12.94	n.d.	**233 ± 43 a**	**647 ± 294 a**	n.d.	n.d.	**212 ± 108 a**	**447 ± 68 a**	n.d.	F_3,8_ = 0.88; *p* = 0.488
**E2HZ3H**	12.99	n.d.	**302 ± 53 a**	**983 ± 518 a**	n.d.	n.d.	n.d.	n.d.	n.d.	F_1,4_ = 0.53; *p* = 0.506
**E2HE2H**	13.45	n.d.	**758 ± 202 a**	**2274 ± 1375 a**	n.d.	n.d.	n.d.	n.d.	n.d.	F_1,4_ = 1.56; *p* = 0.293
**MI**	17.19	n.d.	n.d.	n.d.	**2214 ± 436**	n.d.	n.d.	n.d.	n.d.	-

RT: Retention time; E2HH: (*E*)-2-hexenyl hexanoate; E2HZ3H: (*E*)-2-hexenyl (*Z*)-3-hexenoate; E2HE2H: (*E*)-2-hexenyl (*E*)-2-hexenoate; MI: myristyl isobutyrate. MTGs: metathoracic scent glands. Data were analyzed using generalized linear models (GLMs) with a Gaussian error distribution. For each compound, pairwise comparisons were performed among each tissue (head, thorax, MTGs, and abdomen) and two sexes by using the *emmeans* package in R. (#) Different lowercase letters indicate statistical differences among tissues of males and females for each compound (*p* < 0.05). Bold numbers indicate the highest amount of the compound in a specific tissue of either sex. “trace” indicates that the compound was detected at a low amount. n.d. indicates that the compound was not detected. (Δ): The MTGs had been removed for the thoraxes.

## Data Availability

All data used in this manuscript were archived on Mendeley Data at https://data.mendeley.com/datasets/mp3n28h4sf/1 (accessed on 20 April 2026) (DOI: 10.17632/mp3n28h4sf.1).
